# Proximal femoral replacement for non-neoplastic conditions: a systematic review on current outcomes

**DOI:** 10.1186/s10195-022-00632-z

**Published:** 2022-03-29

**Authors:** Alberto Di Martino, Davide Pederiva, Barbara Bordini, Gabriele Di Carlo, Alessandro Panciera, Giuseppe Geraci, Niccolò Stefanini, Cesare Faldini

**Affiliations:** 1 Department of Biomedical and Neurimotor Sciences - University of Bologna, Piazza di Porta S. Donato, 2, 40127 Bologna, Italy; 2 Ist Orthopaedic Department, IRCCS – Istituto Ortopedico Rizzoli, via Giulio Cesare Pupilli, 1, 40136 Bologna, Italy; 3 Medical Technology Lab, IRCCS – Istituto Ortopedico Rizzoli, Via Giulio Cesare Pupilli, 1, 40136 Bologna, Italy

**Keywords:** Megaprosthesis, Proximal femoral replacement, Hip arthroplasty, Revision surgery

## Abstract

Proximal femoral replacement (PFR) is a well-established treatment for neoplasia of the proximal femur. The use of this surgical technique for non-neoplastic conditions has increased over the years. We carried out a systematic review of the literature to study the indications, complications, and functional results when PFR is used for non-neoplastic conditions. Twenty-seven studies were included in the review with a total of 828 PFRs with a mean follow-up of 50 months (range 1–225 months). The main indications were infection (28%), periprosthetic fracture (27%), aseptic loosening (22%), and fracture (16%). The rate of reoperation was 20.3% overall. The overall revision rate was 15.4%. The main complications were dislocation (10.2%) and infection (7.3%). After 2010, the rates of reoperation (25.5% versus 18.2%), loosening (9.4% versus 3.2%), and dislocation (15.7% versus 7.9%) were lower than before 2010. The 30-day mortality ranged from 0% to 9%. The hip function scores improved post-surgery. In conclusion, the use of PFR in non-neoplastic conditions remains a marginal tool, associated with low direct mortality and high complication rates, but we expect its use to increase in the near future.

## Introduction

The increase in the number of total hip arthroplasties (THAs) performed, also in younger and more active patients, has led to an increase in the number of revision surgeries [1–5]. Factors associated with revision THA, such as periprosthetic fractures, osteolysis, infections, and multiple surgical operations, contribute to bone stock loss of the proximal femur, which creates a challenge in femoral component positioning [6–8].

Treatment options mainly include the use of long revision stems, allograft-prosthesis composites (APC), and proximal femur replacement (PFR). However, when these options are not suitable solutions because of poor bone or tissue conditions, even more invasive procedures such as total femur replacement (TFR) and resection arthroplasty (RA) have been proposed [9–15].

**Fig. 1:**
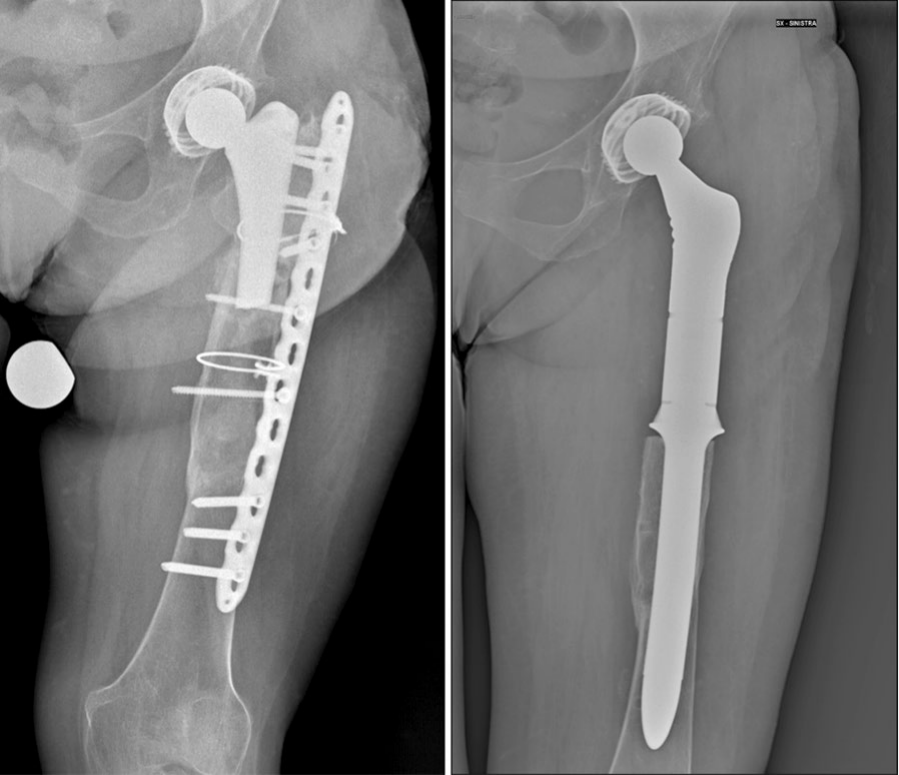
Pre- and postoperative radiographies of a patient with periprosthetic and prosthesis component fracture treated first with plate and cable fixation and then, because of persistent pain and unstable fixation, with proximal femoral replacement

PFR, developed initially as a tool for the management of neoplastic diseases, has slowly gained a role in the treatment of non-neoplastic conditions as a salvage procedure in patients with severe bone loss at the proximal femur (Fig. 1) [16–18].

PFR has some peculiar pros and cons. Two advantages are the possibility of early weight bearing since no bone healing is required and the avoidance of some of the complications associated with allograft use, such as risk of infective disease transmission and fractures. The surgical technique is relatively easy to perform, but it is associated with complications, including damage to the abductor hip muscles and an increased rate of infections when compared with primary THAs [9, 19–21].

In experienced hands, it can be a smart solution to achieve reconstruction and fast return to weight bearing in fragile patients, often after failure of previous surgeries [19, 22–25].

Publications that specifically target the use of PFR for non-neoplastic conditions are scarce, with the last systematic review on the topic published in 2014 by Korim et al. [26]. Given the rapid development of new materials and implants and the increased use of PFR in the non-oncologic setting, an up-to-date systematic review of the literature was performed to obtain a comprehensive picture on current surgical indications, complications, and functional results of these implants.

## Materials and methods

### Literature search

The medical PubMed and MEDLINE databases and Cochrane Database of Systematic Reviews were analyzed on 17 November 2020, searching for relevant publications on the use of PFRs for non-neoplastic conditions. The databases were filtered for studies published between January 1980 and November 2020, in English language. The keywords used for the search were “Proximal femoral replacement” and “Hip megaprothesis.” References of relevant papers were then analyzed to find additional works pertinent to the topic.

Articles describing the use of proximal femoral replacement for the management of non-neoplastic conditions, published in English, were included. Conversely, articles were excluded if describing the use of proximal femoral replacement for the management of neoplastic pathology, if published in a language other than English, if regarding patients operated on for TFR, or if describing outcomes of fewer than five patients [26]. Two studies [24, 27], despite being very informative, were excluded because a distinction between proximal and distal femoral replacements could not be made.

The titles and abstracts of the identified papers were analyzed and screened independently by two authors. The articles judged of interest were then selected for a full text analysis according to our inclusion and exclusion criteria. The Preferred Reporting Item for Systematic Reviews and Meta-Analyses (PRISMA) guidelines [28] were followed, and a flowchart was created to summarize the inclusion process of the analyzed works.

### Qualitative assessment

The level of evidence and the quality of each study was assessed by the methodological index for non-randomized studies (MINORS) [29] by two authors independently. It explores 12 elements, the first 8 developed to specifically evaluate quality of research in nonrandomized studies. These include clear statement of the study aim, inclusion of consecutive patients, prospective data collection, and appropriateness of the endpoint to the aim of the study, which should be unbiased; moreover, the follow-up of the study should be clearly reported as being appropriate for the aim of the study, with loss of less than 5% of patients at follow-up. Finally, a prospective calculation of the study size should be clearly stated in the material and methods section of the study. Each element is given a score from 0 to 2: 0 if not reported, 1 if reported but inadequate, 2 if reported and adequate. The ideal overall score for noncomparative studies is 16.

### Data extraction and report

The selected studies were analyzed, and the information of interest was extracted onto a database created using Microsoft Excel for Microsoft Mac (Microsoft Corporation, Redmond, Washington, USA).

The following data were recorded, if available: number of patients, age of patients, number of previous surgeries, length of follow-up, performed surgical approach, type of implant used at index surgery, type of fixation including the use of bone cement, use of a constrained liner, indications to surgery, intra- and perioperative mortality (within 30 days from surgery), reoperation rate, revision rate (intended as the exchange of any of the prosthesis’ components), postoperative complications (loosening, dislocation, periprosthetic fracture, infection, hematoma, wound healing problems), and hip functional scores. The extracted data were reported with the use of descriptive statistics. Continuous variables were reported as average and range (minimum–maximum). Categorical variables were reported as frequencies and percentages.

### Outcome measures

The primary outcomes were the surgical indications for the use of PFRs and the surgical approach used for these implants.

The secondary outcomes were the failure rate and 30-day mortality. Failure was defined as the need for reoperation, due to mechanical (subclassified as loosening, dislocation, fracture of prosthesis component, or periprosthetic fracture) or nonmechanical reasons (subclassified as infection, hematoma, or wound dehiscence needing incision and drainage), according to the classifications used in the last systematic review on the topic [26]. Surgeries requiring partial or total implant replacement were recorded and reported as revisions.

The tertiary outcome was the comparison of the hip functioning scores in the pre- and postoperative period.

### Statistical analysis

The primary and secondary outcomes were further divided according to the date of publication (before versus after 1 January 2010), and the extracted data were compared with the use of chi-squared test to identify statistically relevant differences. Chi-squared tests of independence were performed to determine whether the proportions of one nominal variable were different for different values of the other nominal variable.

The null hypothesis was that the relative proportions of one variable were independent of the second variable. If chi-squared test was significant, the data were further investigated using pairwise comparisons with Bonferroni corrections of the *P* values. Statistical significance was set at *P* < 0.05.

## Results

### Search results

**Fig. 2:**
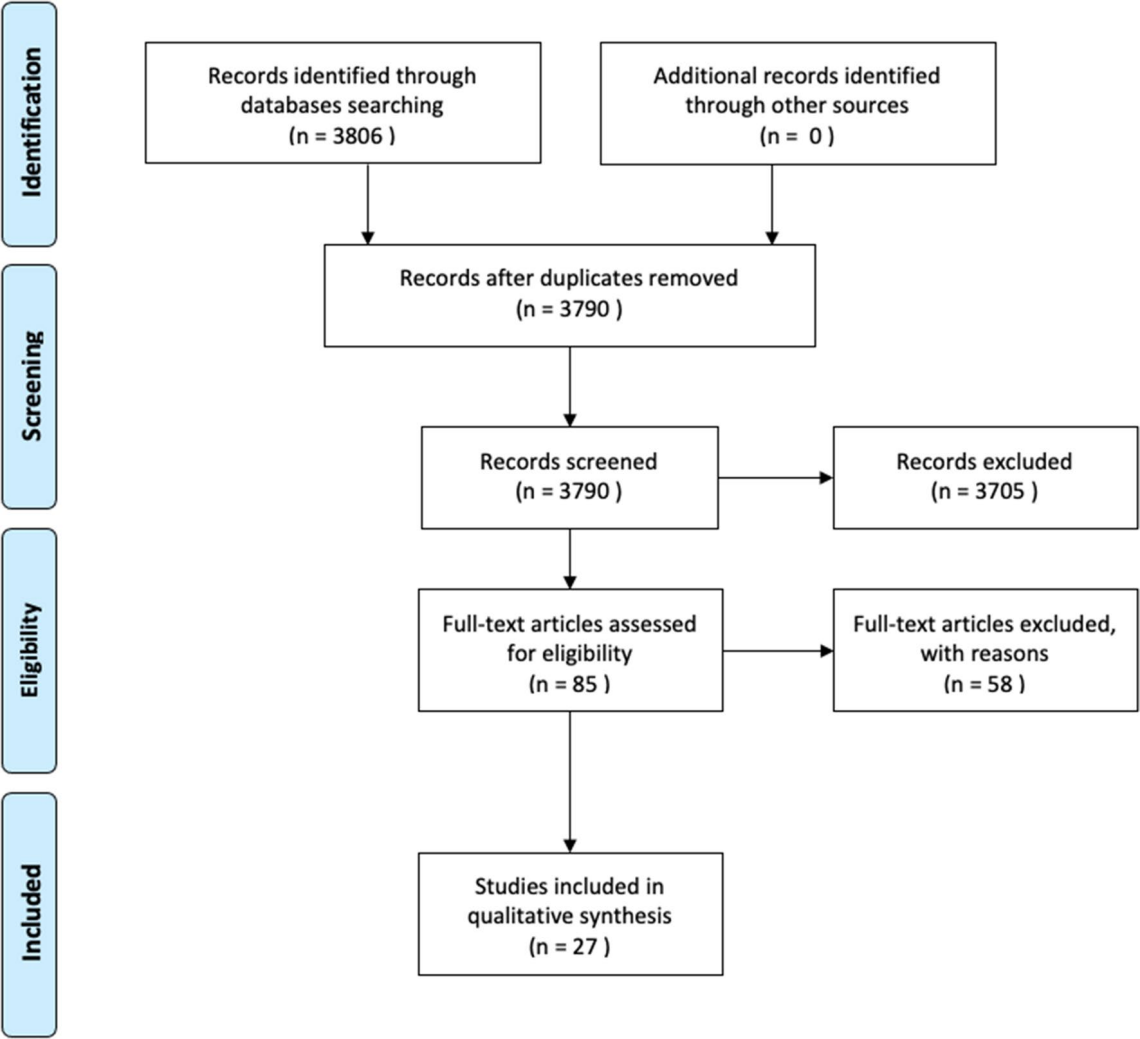
PRISMA flowchart showing the search for and selection of papers

The study selection process is summarized in Fig. 2, using the PRISMA flowchart. The initial search yielded 3806 papers. On the basis of title and abstract, 3705 studies were excluded because they were considered not relevant to the present study. The remaining 85 papers were evaluated in detail to verify the congruence with the inclusion criteria. After full text screening, a total of 27 studies met the inclusion criteria.

### Study quality assessment

All the selected studies were retrospective case series of small to medium size (*N* = 5–80) reporting the outcomes of the use of PFR for non-neoplastic conditions. Manuscripts were published between 1980 and 2020. According to the MINORS evaluating score system, 2 studies reported 6/16 points [30, 31], and another 2 reported 7/16 points [11, 32]; 15 studies achieved 8/16 points [16, 19, 20, 22, 23, 33–42], 1 achieved 10/16 points [10], and 7 had a score of 12/16 [17, 18, 21, 43–46].

### Cohort characteristics

The studies included 828 patients (Table 1) with a mean age of 70.9 years (minimum 22 years to maximum 102 years); average follow-up was 50 months (1–225 months). Patients had an average of 2.6 surgeries before PFR, with four patients having the implant at first surgery, and one patient after 22.

**Table 1:** Cohort characteristics

Study	*N*	Mean age (range), years	Mean follow-up (range), months	Mean prior surgeries (range)
Bosquet et al., 1980 [30]	7	66.7 (40–78)	33 (9–66)	–
Sim and Chao, 1981 [32]	21	59.3 (28–77)	43 (25–92)	2.5 (1–6)
Malkani et al., 1995 [16]	50	60.6 (27–82)	133 (61–225)	–
Haentjens et al., 1996 [31]	19	78.0 (63–87)	60 (24–132)	(1–6)
Klein et al., 2005 [22]	21	78.3 (52–90)	38 (24–84)	3 (1–7)
Shih et al., 2007 [33]	12	59.0 (25–75)	68 (40–108)	6.5 (3–22)
Parvizi et al., 2007 [17]	48	73.8 (42–97)	36 (24–79)	2.7 (0–8)
Jaiswal et al., 2008 [34]	27	68.4 (50–84)	55 (25–126)	2.2 (1–4)
Schoenfeld et al., 2008 [35]	22	74.6 (54–90)	44 (2–132)	–
Bertani et al., 2009 [36]	8	65	65/44	–
Subtotal before 2010	235	68.6 (25–97)	65 (2–225)	
Gebert et al., 2010 [37]	45	62.2 (31–81)	38 (12–70)	–
Al-Taki et al., 2011 [10]	63	73.0 (23–94)	38 (24–120)	–
Dean et al., 2012 [38]	8	67.5 (50–79)	16 (6–36)	2 (1–11)
McLean et al., 2012 [20]	20	72.0 (36–91)	48 (12–116)	–
Colman et al., 2014 [43]	21	75	15	–
Calori et al., 2014 [39]	21	68.0 (30–89)	18 (6–72)	–
Patel et al., 2014 [40]	5	68.2 (59–76)	41 (6–59)	–
Lundh et al., 2014 [23]	5	86.0 (81–91)	29 (13–70)	–
Grammatopoulos et al., 2016 [11]	80	68.0 (28–93)	60 (1–138)	2.4 (0–17)
Viste et al., 2017 [19]	44	79.0 (53–79)	72 (24–144)	3 (1–10)
Corona et al., 2018 [44]	14	75.9 (41–87)	44 (18–82)	–
Khajuria et al., 2018 [45]	37	80.0 (49–94)	33 (6–84)	2.5 (1–9)
Alvand et al., 2018 [21]	40	68.0 (43–92)	46 (24–120)	3.1 (1–10)
De Martino et al., 2019 [41]	41	64.0 (29–90)	60 (24–120)	3.6 (1–11)
Fahad et al., 2019 [42]	21	74.0 (64–91)	33 (8–91)	–
Dieckmann et al., 2019 [46]	49	71.0 (37–85)	52 (6–162)	–
Fenelon et al., 2020 [18]	79	78.3 (46–102)	35 (0–90)	1.4 (0–10)
Subtotal after 2010	593	71.9 (23–102)	44 (0–162)	
Total	828	70.9 (23–102)	50 (1–225)	2.6 (0–22)
Range	5–80	59.3–80.0	15–133	–

### Indications and surgical technique

The most frequent indication for PFR in non-neoplastic conditions (Table 2) was infection (28%, 234/828 patients), followed closely by periprosthetic fracture (27%, 225/828 patients) and aseptic loosening (22%, 186/828 patients). Primary fractures accounted for 16% of cases (135/828 patients) and other conditions for 6% (48/828).

**Table 2:** Indications and surgical technique

Study	*N*	Indication					Surgical technique			
		Fracture, *N* (%)	Periprosthetic, *N* (%)	Aseptic loosening, *N* (%)	Infection, *N* (%)	Other, *N* (%)	Implant	Approach	Cemented (%)	Constrained liner, *N* (%)
Bosquet et al., 1980 [30]	7	0 (0)	3 (42)	2 (29)	0 (0)	2 (29)	A-O	–	C (100)	–
Sim and Chao, 1981 [32]	21	7 (33)	6 (29)	1 (5)	0 (0)	7 (33)	Custom	L	C (100)	–
Malkani et al., 1995 [16]	50	15 (30)	3 (6)	26 (52)	0 (0)	6 (12)	Monobloc	L	C (100)	–
Haentjens et al., 1996 [31]	19	0 (0)	0 (0)	19 (100)	0 (0)	0 (0)	Protek	PL	C (100)	–
Klein et al., 2005 [22]	21	0 (0)	21 (100)	0 (0)	0 (0)	0 (0)	Stryker	L	C (100)	7 (33)
Shih et al., 2007 [33]	12	1 (8)	0 (0)	3 (25)	8 (67)	0 (0)	Custom	P	C (100)	–
Parvizi et al., 2007 [17]	48	1 (2)	20 (42)	0 (0)	13 (27)	14 (29)	MRS	L	C (100)	15 (31)
Jaiswal et al., 2008 [34]	27	0 (0)	5 (19)	22 (81)	0 (0)	0 (0)	Stanmore	L	C (100)	–
Schoenfeld et al., 2008 [35]	22	22 (100)	0 (0)	0 (0)	0 (0)	0 (0)	Mixture	P	C (100)	–
Bertani et al., 2009 [36]	8	0 (0)	1 (12)	7 (88)	0 (0)	0 (0)	JVC-IX	AL	C (100)	–
Subtotal before 2010	235	46 (20)	59 (25)	80 (34)	21 (9)	29 (12)				
Gebert et al., 2010 [37]	45	0 (0)	9 (20)	20 (44)	16 (36)	0 (0)	MUTARS	–	C (7)	–
Al-Taki et al., 2011 [10]	63	0 (0)	27 (43)	27 (43)	7 (11)	2 (3)	MRS/GMRS	AL/PL	C (92)	23 (36)
Dean et al., 2012 [38]	8	6 (75)	0 (0)	0 (0)	2 (25)	0 (0)	METS	–	–	–
McLean et al., 2012 [20]	20	4 (20)	9 (45)	0 (0)	7 (35)	0 (0)	GMRS	L	C (100)	7 (35)
Colman et al., 2014 [43]	21	0 (0)	21 (100)	0 (0)	0 (0)	0 (0)	GMRS	L/AL	C (100)	7 (33)
Calori et al., 2014 [39]	21	12 (57)	3 (14)	6 (29)	0 (0)	0 (0)	–	–	–	–
Patel et al., 2014 [40]	5	5 (100)	0 (0)	0 (0)	0 (0)	0 (0)	Custom	P	C (100)	–
Lundh et al., 2014 [23]	5	0 (0)	5 (100)	0 (0)	0 (0)	0 (0)	METS	P	C (100)	–
Grammatopoulos et al., 2016 [11]	80	12 (15)	12 (15)	6 (7)	40 (50)	10 (12)	Stanmore	PL/L	C (100)	3 (4)
Viste et al., 2017 [19]	44	0 (0)	15 (34)	16 (36)	12 (28)	1 (2)	GMRS	L	C (100)	23 (52)
Corona et al., 2018 [44]	14	0 (0)	0 (0)	0 (0)	14 (100)	0 (0)	Mixture	–	C (64)	–
Khajuria et al., 2018 [45]	37	15 (41)	8 (22)	8 (22)	4 (11)	2 (5)	METS	P	C (100)	–
Alvand et al., 2018 [21]	40	0 (0)	0 (0)	0 (0)	40 (100)	0 (0)	METS	–	C (100)	–
De Martino et al., 2019 [41]	41	3 (7)	7 (17)	14 (34)	17 (41)	0 (0)	GMRS	PL	C (90)	–
Fahad et al., 2019 [42]	21	21 (100)	0 (0)	0 (0)	0 (0)	0 (0)	–	P/L	C (0)	–
Dieckmann et al., 2019 [46]	49	0 (0)	0 (0)	0 (0)	49 (100)	0 (0)	MUTARS	–	C (100)	–
Fenelon et al., 2020 [18]	79	11 (14)	50 (63)	9 (11)	5 (6)	4 (5)	GMRS/LPS	P/AL	C (100)	–
Subtotal since 2010	593	89 (15)	166 (28)	106 (18)	213 (36)	19 (3)				
Total	828	135 (16)	225 (27)	186 (22)	234 (28)	48 (6)				
Range	5–80	0–100%	0–100%	0–100%	0–100%	0–33%				

The most frequently used implants were MRS and GMRS (32%, 261/828), followed by Stanmore (13%, 107/828), MUTARS (11%, 94/828), and METS (11%, 90/828). The implant was fixed with the use of acrylic cement in 90.4% of patients. A constrained liner was used in 10.3% (85/828) of patients.

Surgery was performed by the direct lateral approach in 32% of patients (268/828), followed by the posterolateral (18%, 148/828), posterior (16%, 132/828), and anterolateral (8%, 69/828) approaches. Surgical approach was not reported in 25% of patients (211/828).

Indications to surgery evolved overtime, with aseptic loosening more common in manuscripts published before 2010 (34% versus 18%; *p* < 0.0001); conversely, infection has been a more frequent indication in studies since 2010 (36% versus 9%; *p* < 0.0001). No significant differences were found for surgical indications in trauma patients operated on for fractures (*p* = 0.109) and periprosthetic fractures (*p* = 0.4).

### Complications

One study [10] described the postoperative course of 36 out of 63 total patients, leaving a total of 801 patients for analysis. Reoperations were performed in 163/801 (20.3%) patients (Table 3).

**Table 3:** Complications

Study	*N*	30-day mortality (%)	Reoperations, *N* (%)	Revision, *N* (%)	Mechanical				Nonmechanical	
					Loosening, *N* (%)	Dislocation, *N* (%)	Component fracture, *N* (%)	Periprosthetic number, *N* (%)	Infection, *N* (%)	Wound healing/hematoma, *N* (%)
Bosquet et al., 1980 [30]	7		0 (0)	0 (0)	1 (0)	0 (0)	0 (0)	0 (0)	0 (0)	0 (0)
Sim and Chao, 1981 [32]	21		3 (14)	2 (10)	0 (0)	3 (14)	1 (5)	1 (5)	1 (5)	0 (0)
Malkani et al., 1995 [16]	50		18 (36)	12 (24)	11 (22)	11 (22)	1 (2)	0 (0)	3 (6)	2 (4)
Haentjens et al., 1996 [31]	19		8 (42)	2 (11)	1 (5)	7 (37)	0 (0)	0 (0)	2 (11)	0 (0)
Klein et al., 2005 [22]	21		2 (10)	2 (10)	1 (5)	2 (10)	0 (0)	1 (5)	0 (0)	2 (10)
Shih et al., 2007 [33]	12		5 (42)	5 (42)	1 (8)	2 (17)	0 (0)	0 (0)	4 (33)	0 (0)
Parvizi et al., 2007 [17]	48		11 (23)	10 (21)	4 (8)	8 (17)	0 (0)	0 (0)	1 (2)	0 (0)
Jaiswal et al., 2008 [34]	27		5 (19)	3 (11)	0 (0)	2 (7)	0 (0)	0 (0)	3 (11)	0 (0)
Schoenfeld et al., 2008 [35]	22	2 (9)	3 (14)	2 (9)	0 (0)	2 (9)	0 (0)	2 (9)	1 (5)	0 (0)
Bertani et al., 2009 [36]	8		5 (62)	4 (50)	3 (37)	0 (0)	0 (0)	1 (12)	1 (12)	0 (0)
Subtotal before 2010	235	2 (0.9)	60 (25.5)	42 (17.9)	22 (9.4)	37 (15.7)	2 (0.9)	5 (2.1)	16 (6.8)	4 (1.7)
Gebert et al., 2010 [37]	45		8 (18)	8 (18)	2 (4)	1 (2)	0 (0)	0 (0)	5 (11)	0 (0)
Al-Taki et al., 2011 [10]	36		6 (17)	5 (14)	2 (5)	3 (8)	0 (0)	0 (0)	1 (3)	0 (0)
Dean et al., 2012 [38]	8		0 (0)	0 (0)	0 (0)	0 (0)	0 (0)	0 (0)	0 (0)	0 (0)
McLean et al., 2012 [20]	20		6 (30)	3 (15)	0 (0)	3 (15)	0 (0)	1 (5)	2 (10)	0 (0)
Colman et al., 2014 [43]	21		8 (38)	5 (24)	0 (0)	4 (19)	0 (0)	0 (0)	4 (19)	0 (0)
Calori et al., 2014 [39]	21		2 (10)	2 (10)	0 (0)	2 (10)	0 (0)	0 (0)	0 (0)	0 (0)
Patel et al., 2014 [40]	5		1 (20)	1 (20)	1 (20)	0 (0)	0 (0)	0 (0)	0 (0)	0 (0)
Lundh et al., 2014 [23]	5		0 (0)	0 (0)	0 (0)	2 (40)	0 (0)	0 (0)	0 (0)	1 (20)
Grammatopoulos et al., 2016 [11]	80	1 (1)	17 (21)	12 (15)	3 (4)	3 (4)	0 (0)	5 (6)	9 (11)	0 (0)
Viste et al., 2017 [19]	44		9 (20)	8 (18)	1 (2)	6 (14)	0 (0)	0 (0)	2 (4)	1 (2)
Corona et al., 2018 [44]	14		2 (14)	2 (14)	0 (0)	2 (14)	0 (0)	0 (0)	2 (14)	0 (0)
Khajuria et al., 2018 [45]	37		3 (8)	3 (8)	0 (0)	1 (3)	0 (0)	0 (0)	2 (5)	0 (0)
Alvand et al., 2018 [21]	40		15 (37)	9 (22)	1 (2)	0 (0)	0 (0)	5 (12)	7 (17)	1 (2)
De Martino et al., 2019 [41]	41		8 (19)	7 (17)	2 (5)	2 (5)	0 (0)	2 (5)	3 (7)	0 (0)
Fahad et al., 2019 [42]	21	1 (5)	0 (0)	0 (0)	0 (0)	1 (5)	0 (0)	0 (0)	1 (5)	0 (0)
Dieckmann et al., 2019 [46]	49	1 (2)	14 (29)	12 (24)	6 (12)	6 (12)	0 (0)	1 (2)	2 (4)	6 (12)
Fenelon et al., 2020 [18]	79	6 (8)	4 (5)	4 (5)	0 (0)	9 (11)	0 (0)	0 (0)	3 (4)	0 (0)
Subtotal since 2010	566	9 (1.6)	103 (18.2)	81 (14.3)	18 (3.2)	45 (7.9)	0 (0)	14 (2.5)	43 (7.6)	9 (1.6)
Total	801	11 (1.4)	163 (20.3)	123 (15.4)	40 (5.0)	82 (10.2)	2 (0.2)	19 (2.4)	59 (7.4)	13 (1.6)
Range	5–80	0–9%	0–62%	0–50%	0–37%	0–40%	0–5%	0–12%	0–33%	0–20%

The most frequently reported postoperative complication was dislocation (10.2%, 82/801), followed by infection (7.4%, 59/801) and aseptic loosening (5.0%, 40/801). The fracture of prosthetic components was a rare event occurring in only two patients (0.2%).

Of the 82 dislocations, 38 (46%) underwent a closed reduction, whereas 54% underwent an open reduction during which liner and/or head was exchanged to either a constrained liner or a bigger-sized head.

Of the 59 infections, antibiotic-only treatment was used in five cases (8%). In 24 patients (41%), a debridement of the infected prosthesis was performed, retaining the original components. Twenty-four patients (41%) underwent a two-stage revision in which the prosthetic components were first explanted and then re-implanted after resolution of the infective process. Four patients (7%) underwent resection arthroplasty, and in one patient a conversion to total femur replacement was performed (2%). One patient died because of infection.

Of the 39 patients in whom an aseptic loosening occurred, 29 (74%) were revised for failure of the acetabular or femoral component, and 3 were revised by an APC implant. In four patients (10%) an RA was performed, and three patients (8%) were operated on for conversion to TFR.

Of the 19 periprosthetic fractures, only 4 were treated conservatively. The rest underwent osteosynthesis (21%), revision of the prosthetic components (53%), or conversion to TFR (5%).

Overall, the PFR was revised in 163 (15.4%) patients. The 30-day mortality ranged from 0% to 9%, with an average of 1.4% (11/801).

Reoperations were more frequent in cohorts reported before 2010 compared with reports since 2010 (25.5% versus 18.2%; *p* = 0.021); main causes of revision surgery were mechanical loosening (9.4% versus 3.2%; *p* = 0.0003) and dislocations (15.7% versus 7.9%; *p* = 0.0009). No significant differences were found regarding 30-day mortality (*p* = 0.523), rate of revision (*p* = 0.236), periprosthetic fracture (*p* = 0.770), infection (*p* = 0.697), or wound healing problems (*p* = 0.909).

### Hip function scores

Function scores were reported in 21/27 studies [10, 11, 16, 17, 19–22, 31–38, 40, 42, 44–46] (Table 4), generally showing an improvement from preoperative to postoperative. The most frequently used score was the Harris Hip Score (HHS) [47], followed by the Oxford Hip Score [48]. In the studies where the HHS was used, there was an average improvement of 33 points between preoperative (average 40) and postoperative (average 73) scores.

**Table 4:** Hip scores: 1 = Harris Hip Score; 2 = Musculoskeletal Tumor Society Score [49]; 3 = Oxford Hip Score; 4 = Toronto Extremity Salvage Score [50]; 5 = Lower Extremity Activity Score [51]; 6 = 1 very good, 8 good, 5 fair, 2 poor; 7 = 4 excellent, 3 very good, 4 good, 5 fair, 2 poor, 2 bad

Study	*N*	Hip score before	Hip score after
Bosquet et al., 1980 [30]	7	–	–
Sim and Chao, 1981 [32]	21	–	85 (63–98)^1^
Malkani et al., 1995 [16]	50	46 (31–83)^1^	80 (50–91)^1^
Haentjens et al., 1996 [31]	19	–	MDA^6^
Klein et al., 2005 [22]	21	–	71 (56–90)^1^
Shih et al., 2007 [33]	12	30 (16–42)^1^	83 (68–92)^1^
Parvizi et al., 2007 [17]	48	37^1^	65^1^
Jaiswal et al., 2008 [34]	27	30 (5–58)^1^	72 (44–100)^1^
Schoenfeld et al., 2008 [35]	22	–	MDA^7^
Bertani et al., 2009 [36]	8	–	13.8/6.8^2^
Gebert et al., 2010 [37]	45	30 (8–63)^1^	78 (57–95)^1^
Al-Taki et al., 2011 [10]	36	34.9^3^	54.9^3^
Dean et al., 2012 [38]	8	–	71 (64–85)^1^
McLean et al., 2012 [20]	20	–	68 (32–98)^4^
Colman et al., 2014 [43]	21	–	–
Calori et al., 2014 [39]	21	–	–
Patel et al., 2014 [40]	5	–	24 (21–27)^2^
Lundh et al., 2014 [23]	5	–	–
Grammatopoulos et al., 2016 [11]	80	–	28 (4–48)^3^
Viste et al., 2017 [19]	44	43 (26–83)^1^	68 (21–88)^1^
Corona et al., 2018 [44]	14	–	31 (12–46)^5^
Khajuria et al., 2018 [45]	37	8 (0–16)^3^	31 (19–40)^3^
Alvand et al., 2018 [21]	40	–	22 (4–39)^3^
De Martino et al., 2019 [41]	41	–	–
Fahad et al., 2019 [42]	21	68^1^	66.5^1^
Dieckmann et al., 2019 [46]	49	–	69 (40–94)^1^
Fenelon et al., 2020 [18]	79	–	–
Total	801		

## Discussion

The use of PFR for non-neoplastic conditions remains a marginal surgical tool. In the current systematic review, the main indications for surgery are infection (28%), periprosthetic fracture (27%), and aseptic loosening (22%). The most frequent complications are dislocation (10.2%) and infection (7.3%) with an overall reoperation rate of 20.3%. The 30-day mortality ranges between 0% and 9%. Interestingly, a reduced rate of complications in studies published after 2010 was found. In particular, there were significantly fewer reoperations for any reason (18.2% versus 25.5%), fewer loosenings (3.2% versus 9.4%), and fewer dislocations (7.9% versus 15.7%). Conversely, no differences in infection rates before or after 2010 were observed (6.8% versus 7.6%) despite the significant prevalence in infection as an indication to PFR after 2010 (36% versus 9% of patients). A nonsignificant trend toward fewer implant revisions has been observed in studies since 2010 (14.3% versus 17.9%).

The main limitations of the present study are connected to the low level of evidence and quality of the included manuscripts. In many studies, the sample size is extremely small and the follow-up is not long enough to correctly include the whole clinical history of the patients. A systematic review of the subject, by pooling the data together, can overcome the limitation of small sample size. Moreover, the time range of published manuscripts spans over almost 40 years, during which the surgical techniques and technologies have evolved, possibly creating distortions in the obtained results. For this reason, a further analysis on primary and secondary outcomes of literature before and after 2010 was performed.

The common characteristic of all the patients involved in the included studies was the extensive poor quality of the proximal femoral bone stock, mostly described as type IIIA and IIIB according to the classification of Della Valle and Paprosky [52]. Because of such deficiency in case of primary fracture, periprosthetic fracture, component loosening, or infection, the available treatment options are quite scarce, and mainly include PFR and APC. Only in some favorable cases is the use of revision stems alone advisable in this setting, and the outcomes connected to their use are associated with an overall reduction in complication rates compared with data of PFR reported in the current review (Table 5).

**Table 5:** Outcomes of different surgical techniques for the management of patients with poor quality of proximal femoral bone stock

Surgical technique	Complications								HHS pre–post difference
	Direct mortality	Reoperation	Revision	Dislocation	Infection	Aseptic loosening	Component fracture	Periprosthetic fracture	
PFR	1.4%	20.3%	15.4%	10.2%	7.4%	5.0%	0.2%	2.4%	+33
Revision stem	2.3% [53]	11% [54]	13.2% [55]; 5.9% [53]	1.0% [54]; 3.0% [55]; 7.8% [53]	0.5% [54]; 2.9% [55]; 4.9% [53]	1.3% [54]; 3.1% [55]; 5.8% [53]	1.7% [56]; 2.3% [57]	0.5% [54]; 3.1% [53]	+37 [53]
APC	0% [58]	60% [59]	16.8% [13]; 26.5% [59]; 14% [60]	12.8% [13]; 11.1% [59]; 8% [60]	6.9% [59]; 4% [60]	5.5% [59]; 12% [60]	1.4% [59]	allograft 5.6% femoral 2.8% [59]	+41 [59]; +34 [61]
TFR	0% [62]	33% [63]	21–30% [64]; 18.5% [63]	6–28% [64]; 10% [63]	4–35% [64]; 19% [63]	4% [63]	0–11% [64]	0–1.3% [65]	+14–35 [64]
Resection arthroplasty	10% [66]	8% [66]	–	–	15% [66]	–	–	–	Pain relief 85–100% [67]

Weiss et al. [54], in their study reporting the outcome of 1885 revisions managed by a long revision stem, found an 11% reoperation rate. The main complications were aseptic loosening in 25 (1.3%), dislocations in 19 (1.0%), deep infection in 9 (0.5%), periprosthetic fracture in 9 (0.5%). In a similar study on 9952 revisions cases, re-revisions accounted for 13.2% [55]; reported complications included aseptic loosening (3.1%), dislocation (3.0%), and infection (2.9%). Regarding functional outcomes, Saleh et al. [53] reported the results of 2163 revisions and found an improvement of 37 points from preoperative to postoperative HHS. The reported re-revision rate was 5.9%, dislocations occurred in 7.8%, deep infection in 4.9%, acetabular loosening in 5.8%, and femoral fatigue fracture in 3.1% [53]. However, the use of revision stems is only rarely an option in patients with severe bone loss. When cemented implants are used, the length of the stem, cemented in place, should extend distally by at least two femoral diameters beyond the area of cancellous bone defect to minimize the risk of loosening [68], often not possible because of the entity of bone loss.

Another alternative is the use of allograft, either fresh-frozen or freeze-dried [69] with a long-stemmed revision prosthesis cemented in the allograft, and press fitted in the host femur, the APC. The procedure carries its own complications (Table 5), mainly including fracture or non-union, but has the peculiar advantage of direct tendon sutures at the enthesis on the graft [70]. However, failure is reported in up to 11–20% of patients [69] requiring revision surgery to PFR in most cases, rarely to TFR, or performance of an RA [70].

A review of 498 hips managed with this technique outlines a non-union rate of 25.2% (range 0–77%), a dislocation rate of 12.8% (range 0–55%), and the need for a revision surgery in 16.8% (range 5–34%) [13]. Some authors report even a higher revision rate when the technique is used. Babis et al. [59] evaluated the use of APC in 72 patients with severe bone loss. Subsequent revision was performed in 26.5%, with an overall reoperation rate of 59.7%. These complication rates make the APCs more appropriate for younger patients, in which the chance of graft integration is higher, and in which tendon reconstruction is aimed at a more functional hip [69–71], keeping in mind that PFR is a viable option in most cases of APC failure.

Salvage procedures in these patients may require TFR and resection arthroplasty [64, 67]. TFR is usually performed when there is less than 120–130 mm of intact diaphyseal or distal femoral bone available, making it unsuitable for other reconstruction techniques [14]. It allows a functional improvement of 14–35 HHS points postoperatively [64], but recent reports outlined a failure rate of 21–30% for these implants, with common complications including deep infection in 4–35%, dislocation in 6–28%, structural failure in 9%, and aseptic loosening in 4% [63, 64].

RA is mainly performed in non-ambulating patients or in those in whom a reimplantation is considered too risky or prone to failure when the residual femoral length is such to avoid major shortening of the inferior limb [72]. This technique as a salvage procedure allows pain relief in 85–100%, but it is associated with a wide range of satisfaction rates, from low values like 13% to values as high as 83% [67]. In a recent study of 38 hips managed by RA, the procedure was associated with a 10% death rate, a 15% joint infection rate, an 8% reoperation rate, and a 21% rate of major systemic complication [66]. Both TFR and RA are associated with elevated complication rates, mostly connected to the general health status of the patient, and the invasiveness of the surgery, and should be performed in selected patients for whom alternative methods of reconstruction are not suitable.

In conclusion, the use of PFR in the setting of non-neoplastic conditions remains a limited but useful tool that is expected to increase in the near future. It is a valid option in patients with severe proximal femur bone loss after trauma, and in THA failures due to infections, trauma, or mechanical failure. In recent years, outcomes of PFR have been improving, and in experienced hands, mortality directly related to the surgery is low. However, both the surgeons and the patients should be aware of the rates of complications and reoperations associated with this technique.

In the future, multicenter prospective studies on the indications and long-term results of PFR for non-neoplastic conditions could give a broader and better understanding of their benefits and drawbacks, providing data of the utmost importance to improve patient outcome.

## Data Availability

Every article analyzed to extract the data is present and available on the search engines used.
